# Connecting multimodality in human communication

**DOI:** 10.3389/fnhum.2013.00754

**Published:** 2013-11-08

**Authors:** Christina Regenbogen, Ute Habel, Thilo Kellermann

**Affiliations:** ^1^Department of Psychiatry, Psychotherapy, and Psychosomatics, Medical School, RWTH Aachen University, Aachen, Germany; ^2^JARA Translational Brain Medicine, Jülich/Aachen, Germany

**Keywords:** dynamic causal modeling, finite impulse response, empathy, emotion, prosody, facial expressions, speech content, multimodality

## Abstract

A successful reciprocal evaluation of social signals serves as a prerequisite for social coherence and empathy. In a previous fMRI study we studied naturalistic communication situations by presenting video clips to our participants and recording their behavioral responses regarding empathy and its components. In two conditions, all three channels transported congruent emotional or neutral information, respectively. Three conditions selectively presented two emotional channels and one neutral channel and were thus bimodally emotional. We reported channel-specific emotional contributions in modality-related areas, elicited by dynamic video clips with varying combinations of emotionality in facial expressions, prosody, and speech content. However, to better understand the underlying mechanisms accompanying a naturalistically displayed human social interaction in some key regions that presumably serve as specific processing hubs for facial expressions, prosody, and speech content, we pursued a reanalysis of the data. Here, we focused on two different descriptions of temporal characteristics within these three modality-related regions [right fusiform gyrus (FFG), left auditory cortex (AC), left angular gyrus (AG) and left dorsomedial prefrontal cortex (dmPFC)]. By means of a finite impulse response (FIR) analysis within each of the three regions we examined the post-stimulus time-courses as a description of the temporal characteristics of the BOLD response during the video clips. Second, effective connectivity between these areas and the left dmPFC was analyzed using dynamic causal modeling (DCM) in order to describe condition-related modulatory influences on the coupling between these regions. The FIR analysis showed initially diminished activation in bimodally emotional conditions but stronger activation than that observed in neutral videos toward the end of the stimuli, possibly by bottom-up processes in order to compensate for a lack of emotional information. The DCM analysis instead showed a pronounced top-down control. Remarkably, all connections from the dmPFC to the three other regions were modulated by the experimental conditions. This observation is in line with the presumed role of the dmPFC in the allocation of attention. In contrary, all incoming connections to the AG were modulated, indicating its key role in integrating multimodal information and supporting comprehension. Notably, the input from the FFG to the AG was enhanced when facial expressions conveyed emotional information. These findings serve as preliminary results in understanding network dynamics in human emotional communication and empathy.

## Introduction

Human communication relies on a dynamic information exchange of several communication channels. A successful reciprocal evaluation of social signals serves as a prerequisite for social coherence and empathy. During this perceptive process, information from multiple channels is integrated. For example, when we engage in a conversation with someone and see their worried expression, notice a cracking tone of voice and finally understand by verbal information that something tragic has happened, we base our social inference on the available information—here, from facial expressions, prosody, and speech content. In empathy, according to the contextual appraisal process (De Vignemont and Singer, 2006) emotional cues that are presented can either lead to an immediate empathic reaction (“early appraisal model”) or a slightly delayed reaction. This deferred response is assumed to be due to intermediate steps of modulating an earlier, automatically elicited response (“late appraisal model”). Emotional cues therefore benefit from a congruent contextual embedding so they can be interpreted correctly and initiate an empathic reaction. Underlying are two different concepts that help us create an inner picture of the outside world. By means of “bottom-up” processing, sensations come in from the external world. We hear, see, touch, taste our environment and these sensations are cues to infer phenomenal reality and are automatically processed, even if we do not pay attention to them (Vuilleumier et al., 2001). Parallel to this, “top-down” processing of incoming information takes place which restricts the amount of information and works as a selection filter, but also amplifies certain information, depending on the necessity and appropriateness of that specific piece of information (for an introduction see Desimone and Duncan, 1995). Well-researched in the cognitive domain such as visual attention (Desimone, 1996) top-down efforts can be applied to emotional processes, reappraising and modulating hues of emotions in the information we get (Wright et al., 2008; Lee et al., 2010; Mühlberger et al., 2012).

On a neural level, when faced with emotional multimodal information such as the presented example, bottom-up mechanisms include the stimuli's perception. This takes place in unimodal cortices (Kanwisher et al., 1997) as well as in multi- and supramodal areas which combine inputs and perceive emotionality irrespective of the modality it is presented in (Buckner et al., 2000; Campanella and Belin, 2007; Peelen et al., 2010; Seubert et al., 2010). Top-down influences guide this process and further integrate information.

When we reconsider the anectodal social communication example stated above against the background of our everyday experiences it seems, however, quite unnatural to always have the luxury of perceiving as many as three unequivocal sources of information when inferring someone's mental state. In contrary, quite often, one or several channels are completely non-existent, as in email conversation (no facial expressions, and prosody), or on the telephone (no facial expressions). However, based on previous experiences and given the established effort to build a “good gestalt” (Wertheimer, 1923), our brains fill in information. Over the course of development, we learn how someone usually looks when they have a sad-sounding voice, we can also imagine how someone would sound that we see with tears running down their face but a muted speech.

Recent theoretical advances in the formulation of basic principles stating how the brain might function may be resorted to in order to unite descriptions of top-down and bottom-up processes on a cognitive level and hierarchical feed-back and feed-forward connections on a neural level—albeit maintaining a distinction between the two. In this article, we will refer to these concept(s) as the “Bayesian brain hypothesis” of cortical functioning. In short, Bayesian descriptions of cortical functioning rest upon the hierarchical organization of the neocortex (Maunsell and Van Essen, 1983) where Bayesian surprise is propagated from a lower level to the next higher one. Conversely, predictions about the causes of sensory inputs (inferences) are passed from higher levels to lower levels (Mumford, 1992). Deviations from these predictions have been referred to as (Bayesian) surprise (Itti and Baldi, 2009), prediction error (Rao and Ballard, 1999) or free energy (Friston, 2010). Against this background, the brain can be regarded as a Bayesian machine which tries to infer the causes of sensory inputs. In doing so, actual sensory input is tried to be explained by a kind of prior beliefs, expectations or predictions, whilst discrepancies between these expectations and actual inputs are reported from lower levels to higher ones. At each hierarchical stage higher levels try to explain away the prediction errors that are passed by the level below (Friston, 2005). On a cognitive or behavioral level of description the salience or novelty of stimuli can be regarded as examples of such a kind of surprise or prediction error that are assumed to underlie e.g., the startle response or the mismatch negativity (Feldman and Friston, 2010). In a similar vein, effects of incongruence, like those observed in the Stroop task (Stroop, 1935), the Posner paradigm (Posner and Petersen, 1990) or in Simon tasks (Simon, 1969), may have a neural basis which can be characterized as Bayesian, with a hierarchical processing of stimuli and subsequent (motor) responding. When we now return to the topic of multimodal emotional communication, many aspects of the Bayesian brain hypothesis may be considered relevant in experimental situations where different modalities contain different or at least ambiguous emotional connotation. This may also be again linked to the “appraisal model of empathy” (De Vignemont and Singer, 2006) where incongruence may lead to a failure of sufficient embedding of an emotional cue in order to establish successful multimodal integration and/or an unequivocal empathic response.

In a functional magnetic resonance imaging (fMRI) study investigating empathy and its components emotion recognition and affective responses we presented short video clips to our participants (Regenbogen et al., 2012a). These clips presented actors who told short stories and expressed emotions on different communication channels. In two conditions, all channels uniformly transported either only emotional (condition E) or neutral (condition N) information. Three other conditions selectively presented two emotional channels and one neutral channel (conditions “neutral prosody,” E/nP; “neutral facial expression,” E/nF; and “neutral speech content,” E/nC) while a last condition presented unintelligible speech content (condition E/iC). Subjects indicated the actors' emotional valence and their own while fMRI was recorded.

Our findings confirmed the speculation formulated above: multimodal emotionality showed a facilitative effect for empathy, assessed by behavioral ratings of self- and other emotion, and was associated with multimodal integration in thalamus and precuneus. While behavioral empathy clearly decreased when one out of three channels presented non-emotional information neural network activation was still (although weaker) present in regions responsible for inferring someones mental state and understanding their actions (e.g., temporoparietal junction) and in the dorsomedial prefrontal cortex (dmPFC), a region which serves self-referential functioning (Wolf et al., 2010) such that it decouples one's own from other people's perspectives on the self (D'Argembeau et al., 2007; Lamm et al., 2010) and is part of an anterior mentalizing network (Schnell et al., 2011). Further, while neutral facial expressions made it more difficult to recognize the emotion presented in the video clip, neutral speech content mainly influenced participants' ratings of their own affective state (Regenbogen et al., 2012b). This latter finding supports the notion proposed in the late empathic appraisal model (De Vignemont and Singer, 2006). We suggest that emotion-congruent speech content may be necessary for not only recognizing someone else's emotion, but also sharing it, and thus reveal empathy.

**Figure 1:**
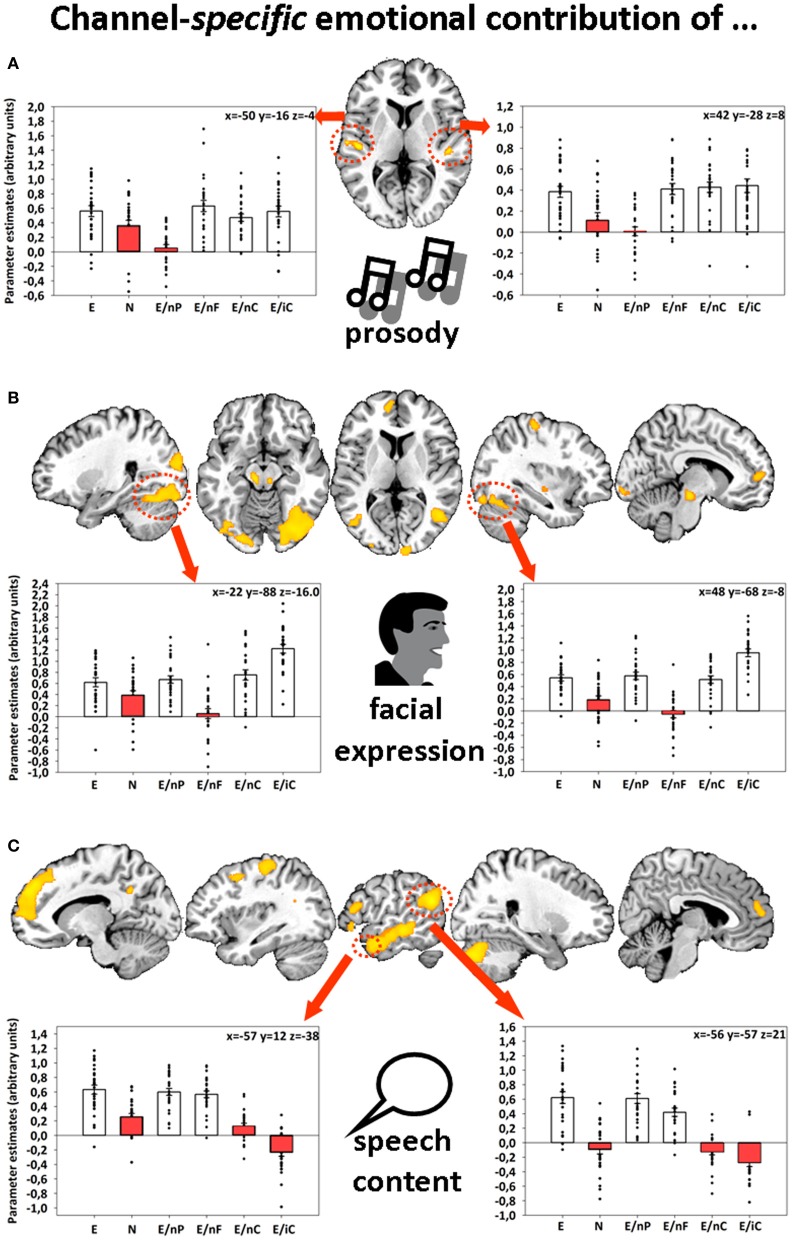
**Sliced MNI brain templates and overlays comprising the group activations of (A) emotional prosody, (B) emotional facial expression, and (C) emotional speech content.** Within several activated clusters (indicated by red dotted shapes), individuals' parameter estimates were plotted onto cluster mean voxel estimates (±*SEM*). MNI coordinates indicate the location of the maximum statistics. Contrast resulted from a random-effects GLM. Re-printed with kind permission of Elsevier (Regenbogen et al., 2012a).

Further, emotions displayed via facial expressions, prosody, and speech content were associated with bilateral activations in fusiform gyri (FFG), auditory cortices (AC), and left angular gyrus (AG), respectively (Figure 1). The identification of these four different network-nodes associated with the perception of human interactions constituted a prerequisite for understanding the dynamics that underlie multimodal integration and at the same time being partly able to explain participants' behavioral empathy decline to incomplete information.

In this reanalysis of the same data set, we pursued two different descriptions of temporal characteristics within these three modality-related regions (FFG, AC, AG) and the dmPFC: First, we examined the post-stimulus time-courses within each of the six conditions because of the prolonged stimulus durations (~10 s) during which different temporal patterns regarding activation levels are likely to occur. This exploratory description of the temporal characteristics of the BOLD response during the videos was accomplished by means of a finite impulse response (FIR) analysis within the FFG, AC, and AG, respectively. Second, the network dynamics in terms of effective connectivity between these areas and the dmPFC were analyzed using dynamic causal modeling (DCM) in order to describe stimulus condition-related modulatory influences on the coupling between these regions.

## Methods

### Previous fMRI study

#### Participants and task

The final sample of the previous fMRI study (Regenbogen et al., 2012a) consisted of 27 right-handed healthy participants (13 females, *M* age = 34.07 years, *SD* = 9.82 years, no history of psychiatric disorder, neurological illness, current substance abuse or lifetime substance addiction) with normal or corrected-to-normal vision and positive MR scanning inclusion criteria.

Subjects were informed about the study protocol, familiarized with the stimulus presentation environment and gave written informed consent. During the fMRI experiment, they were presented with 96 short video clips depicting actors telling a self-related story. After the clip, they were asked to rate the emotional valence of the presented actor, as well as their own (each scaled −3 very negative, +3 very positive) and to indicate this via button press. If this resulted in a matching of other's and own (correct) emotion, the answer was defined as empathic. The experimental set-up was designed according to the Declaration of Helsinki and the study was approved by the local institutional review board.

#### Stimuli, fMRI design and data analysis

Stimuli consisted of 96 thoroughly evaluated video clips (for details please refer to Regenbogen et al., 2012b) with an average duration of 11.8 s (*SD* = 1 s). Clips depicted either a male or a female conversational partner who told self-related stories of different emotional valence (disgust, fear, happiness, sadness, or neutral). Six conditions with 16 videos each (all emotions collapsed) displayed different combinations of prosody, facial expression, and speech content. “All emotional” (E) included emotional stories with congruent emotional facial expression and prosody. “All neutral” (N) contained neutral stories with neutral facial expression and prosody. “Neutral prosody” (E/nP), “neutral face” (E/nF), or “neutral speech content” (E/nC) had the respective channel presented neutral while the two other channels transferred an emotion. “Incomprehensive speech content” (E/iC) consisted of emotional prosody and facial expressions, yet incomprehensive foreign speech content (Polish, Russian, Croatian, Yugoslavian, or Serbian).

Functional images were obtained on a 3 T Trio® MR scanner (Siemens Medical Systems, Erlangen, Germany) during a single session using T2^*^ weighted echo-planar imaging (EPI) sensitive to blood oxygenation level dependent (BOLD) changes (voxel size: 3.125 × 3.125 × 3.1 mm, matrix size: 64 × 64, field of view (FoV): 200 × 200 mm^2^, 36 axial (AC-PC) slices, gap 0.356 mm, TR/TE = 2000/30 ms, flip angle: 76°, 1180 volumes, total duration: 39.33 min).

Data analysis was performed with SPM8 (Wellcome Department of Cognitive Neurology, London). Data preprocessing included realignment, coregistration of the mean functional image into Montreal Neurologic Institute (MNI) space which delivered the priors for a segmentation process in which the mean image was non-linearly segmented into gray matter, white matter, and cerebrospinal fluid (unified segmentation, Ashburner and Friston, 2005). Fitting the mean functional image's gray matter, white matter, and cerebrospinal fluid segments with the tissue probability maps yielded the normalization parameters. These were applied to the time series, including resampling to a voxel size of 1.5 × 1.5 × 1.5 mm. Spatial smoothing on normalized images was carried out with an isotropic 8 mm FWHM (full width at half maximum) Gaussian kernel.

On a single-subject level, one regressor for each condition and one modeling the rating period were created and six realignment parameters were included as covariates of no interest. Serial auto-correlations were accounted for by including a first order autoregressive model. A mixed-effects general linear model (GLM) was used for group-level inference with subjects as random effects and conditions as fixed effects (for both factors heteroscedasticity assumed).

Three channel-specific contrasts were calculated in order to extract the emotional information of one communication channel each: [(E > E/nP) ∩ (E > N^1^)] for emotional prosody, [(E > E/nF) ∩ (E > N)] for emotional facial expression, and [(E > E/nC) ∩ (E > N)] for emotional speech content. To identify brain regions that are activated when emotions are presented through at least two of the three channels a four-fold conjunction analysis (Nichols et al., 2005) analyzed all three conditions with emotion present in 2/3 of the channels compared to the fully natural one [bimodal: (E/nP > N) ∩ (E/nF > N) ∩ (E/nC > N) ∩ (E > N)]. Further details regarding the fMRI experiment can be found in Regenbogen et al. (2012a).

### Finite impulse response (FIR) analysis

The FIR analysis was constrained to regions of interest (ROI) and was performed using the SPM toolbox MarsBaR (http://marsbar.sourceforge.net/). The ROIs were specified based on the peak activation in the respective contrast (“emotional prosody,” “emotional facial expression,” “emotional speech content,” “bimodal”). Time-series were extracted from the left AC (*x* = −50, *y* = −17, *z* = −5), right FFG (*x* = 48, *y* = −68, *z* = −8), left AG (*x* = −56, *y* = −57, *z* = 21) and left dmPFC (*x* = −2, *y* = 21, *z* = 54). Data per ROI were averaged within a sphere of 10 mm radius around these peak coordinates, except for the AC, where the whole supra-threshold cluster was averaged. A window length (time bin) of 2 s was used, which corresponded to the repetition time (TR), and the order was set to 9 (corresponding to 18 s post-stimulus time) so that responses to the whole videos were captured including the expected hemodynamic delay.

The extracted mean values (one for each time-bin, totaling to 9 per subject and condition) were analyzed separately per ROI in IBM® SPSS® (version 20) by means of a two-way repeated measures ANOVA. The two factors were TIME with nine levels referring to the time bins and COND with three levels referring to the type of video. Two levels of the latter factor were identical for all ROIs, namely “all emotional” (E) and “all neutral” (N), while the last level was region-specific, sending neutral information to the respective modality-responsive region: E/nP for the AC, E/nF for the FFG and E/nC for the AG, respectively.

We tested the interaction term TIME x COND for significance and performed two-tailed *post-hoc t*-tests for contrasts which focused on the comparison between the three conditions at each time-point. Because this analysis is rather exploratory and the Bonferroni correction is quite conservative, we decided to correct the threshold for each ANOVA individually [i.e., with (9 − 1) × (3 − 1) = 16 independent comparisons] instead of including the number of ROIs (which would have resulted in a correction factor of 16 × 3 = 48). It must be emphasized that this procedure must be regarded as exploratory, because it does not adhere to the formally correct adjustment. Nevertheless, this approach is more conservative than not correcting for multiple comparisons at all.

### Dynamic causal modeling (DCM) analysis

The regions for the DCM analysis were by name the same as in the FIR analysis. Because DCM infers on the causes of the MR-signal—namely on the hidden neuronal states or their activity induced by the stimuli—only those voxel of a region were included that survived an uncorrected threshold of *p* < 0.01 in an *F*-contrast spanning the columns of interest in the first-level design of each subject. Before subjected to the DCM analysis, the data were adjusted in the sense that they were high-pass filtered (as described in the usual first-level analysis) and the effects of the covariates (intercept and realignment parameters) were removed. In order to address effective connectivity between these regions we used *post-hoc* Bayesian model selection (BMS) as introduced by Friston and Penny (Friston and Penny, 2011). Since we could hardly restrict the potentially huge model space a priori we decided to explore a large model space in a *post-hoc* fashion which is unfeasible for a standard BMS procedure because of its computational burden. Therefore, we applied *post-hoc* BMS by specifying a superordinate, “full” DCM whose subspace was searched under the Laplace approximation to determine an optimized DCM (Friston and Penny, 2011). We assumed an almost full average connectivity structure between the four regions, where just the reciprocal connections between AC and FFG were omitted. Corresponding modulatory connectivities were included for the six different inputs or conditions (see Regenbogen et al., 2012a). These six conditions also served as driving inputs on the two nodes AC and FFG. The ensuing optimized DCMs for each subject were then averaged by means of Bayesian parameter averaging (BPA) as implemented in SPM8.

## Results

### FIR analysis

**Figure 2:**
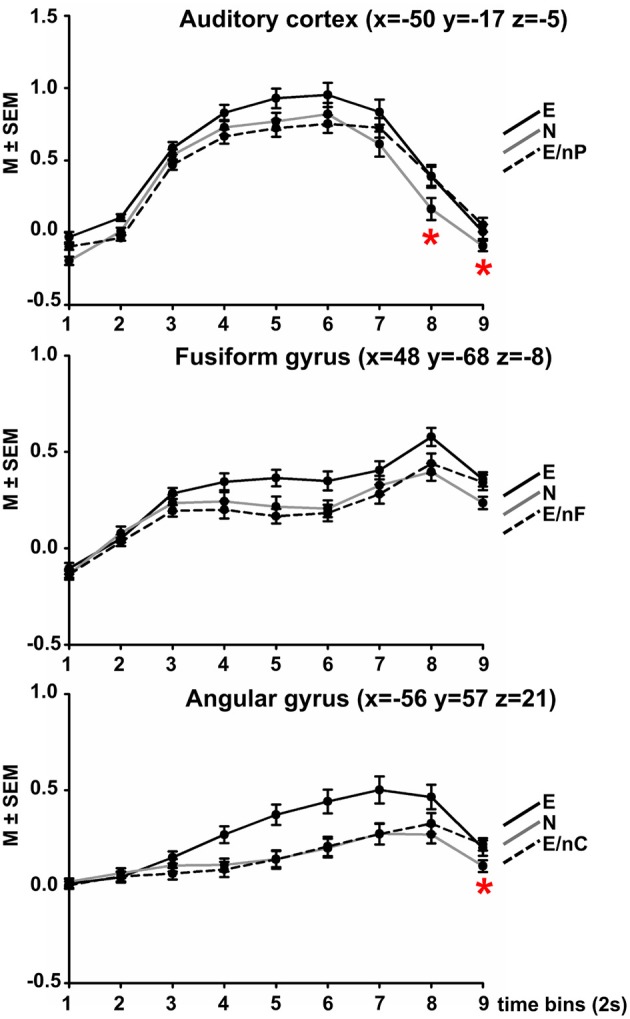
**Results of Finite Impulse Response (FIR) analyses in three modality-responsive regions (AC, FFG, AG, peak coordinate in brackets).** Within each sphere (10 mm around peak amplitude, whole AC, respectively), the time-course of activation within three selected experimental conditions is displayed across 9 time-bins of 2 s each. Black coloring indicates the time-course of the all emotional (E) condition, gray coloring indicates condition all neutral (N), dotted black coloring indicates the bimodally emotional condition in which the input of the modality-responsive region was neutral (E/nP in the AC, E/nF in the FFG, E/nC in the AG). Red asterisks indicate at which time point the respective bimodally emotional condition significantly surpassed the fully neutral condition (N). For all *post-hoc* pairwise comparisons between all three conditions at each time-point please refer to Table S1 in the Supplement.

The ANOVA comparing each nine time-bins (TIME) and three conditions (COND) yielded the following results in each region (Figure 2).

#### Auditory cortex

The ANOVA showed significant main effects of COND [*F*_(2, 52)_ = 27.65, *p* < 0.001], TIME [*F*_(2.16, 56.10)_ = 117.64, *p* < 0.001], and a significant interaction of COND × TIME [*F*_(7.70, 200.16)_ = 9.24, *p* < 0.001].

A complete listing of *post-hoc* comparisons of interest are listed in Table S1 in the Supplement and are Bonferroni-corrected for 16 independent comparisons (corrected threshold *p* = 0.001581). Figure 2 displays the time-course plotted at intervals of 1 TR = 2 s). Condition E surpassed condition N at each time point (significantly at time-points 5, 7, and 9) and also condition E/nP (significantly at time-points 2–6), condition E/nP was actually lowest of the three across the first 6 time-points, even lower than N (n.s.). However, at time-point 7, condition E/nP surpassed condition N and this was significant at time-points 8 and 9.

#### Fusiform gyrus

The ANOVA showed significant main effects of COND [*F*_(1.38, 35.90)_ = 9.92, *p* = 0.001], TIME [*F*_(2.71, 70.44)_ = 45.66, *p* < 0.001], and a significant interaction of COND × TIME [*F*_(8.17, 212.36)_ = 5.36, *p* = 0.002]. For *post-hoc* comparisons please refer to Table S1. Condition E again surpassed condition N at each time point (significantly at time-points 5, 6, 8, and 9) and also condition E/nF (significantly at time-points 4, 5, 6, and 8), condition E/nP was actually lowest of the three across the first 7 time-points, even lower than N (n.s.). However, at time-point 7, condition E/nP surpassed condition N (n.s.).

#### Angular gyrus

The ANOVA showed significant main effects of COND [*F*_(2, 52)_ = 25.31, *p* < 0.001], a significant main effect of TIME [*F*_(1.57, 40.85)_ = 27.65, *p* < 0.001] and a significant interaction of COND × TIME [*F*_(5.64, 146.72)_ = 8.69, *p* < 0.001]. Condition E surpassed condition N at each time point (significantly at time-points 4–8) and also condition E/nC (significantly at time-points 4–8), condition E/nC was lowest of the three across the first 7 time-points (exception for time-point 6 where it was slightly above), even lower than N (n.s.). However, at time-point 8, condition E/nP surpassed condition N (significantly at time-point 9). (Figure 2; Table S1).

### DCM results

**Figure 3:**
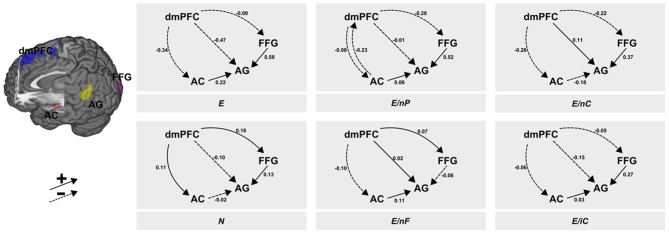
**Results of the DCM analysis.** The six panels show the modulatory inputs of the six different conditions on the connections between the regions. The stimuli conveyed neutral or emotional information via facial expressions, prosody and speech content. These channels were either consistent [all neutral (N) or all emotional (E)] or one of the three channels was neutral whereas the other two transmitted emotional information (E/nP, neutral prosody; E/nF, neutral facial expression; E/nC, neutral speech content). The last condition contained incomprehensible speech (E/iC). AC, auditory cortex; FFG, fusiform gyrus; AG, angular gyrus; dmPFC, dorsomedial prefrontal cortex.

The *post-hoc* BMS showed a clear peak on one model which had a posterior probability of more than 0.99 for which reason we constrain the descriptions to this winning model. The context-dependent changes according to the six experimental conditions in the coupling between regions are illustrated in Figure 3. From visual inspection of the modulatory inputs it is quite striking that always the same connections were affected by one of the six conditions—apart from one exception, namely condition E/nP during which the AC→dmPFC connection was affected in addition to the other ones. Moreover, the general pattern of modulated connections was of the form that all connections *from* dmPFC and all connections *to* AG were affected, albeit the size and even the sign of those modulations differed between conditions. The specific modulatory effects of each of the six conditions on the connections are briefly described in the following paragraphs.

#### All emotional—E

While the dmPFC exerted a negative force (to AC and AG) or minor negative one (to FFG), the input from AC to AG showed a positive input in this (consistently emotional) experimental condition. This positive input was found in all other conditions with emotional speech content, but manifested strongest here. In both conditions with neutral speech content the AC exerted a slightly negative (N) or moderately negative (E/nC) influence. Further, the FFG exerted a strongly positive connection to the AG.

#### All neutral—N

The fully neutral condition lead to an increase of inputs from dmPFC to both, FFG and AC. The input from dmPFC to AG stayed negative as in E but was less pronounced. Condition N (as well as E/nC) resulted in a decrease of connectivity from AC to the AG as it was present in all other conditions with emotional speech content. Further, the increasing modulatory inputs from FFG to AG were smallest in this condition (and negative in condition E/nF) while this connectivity was large in any other condition with an emotional facial expression.

#### Neutral prosody—E/nP

Like in condition E, the dmPFC exerted negative inputs to AC, AG and FFG, although these were small to AC and AG. Further, uniquely present here was a negative input back from AC to the dmPFC. The input from AC to AG was slightly positive and again, the connection from FFG to AG was (strongly) positive.

#### Neutral face—E/nF

When the facial expression was selectively switched to neutral, connectivity from dmPFC to the FFG and to the AG was slightly enhanced. Further, inputs from dmPFC to the AC were slightly decreased. Like in all other conditions with emotional speech content (except N and E/nC) connectivity from AC to AG was positive. This condition was also the only condition in which the input from FFG to AG was not increased but significantly decreased.

#### Neutral speech content—E/nC

When speech content was neutral, connectivity from dmPFC to the AG was increased. This was also the case in condition E/nF. Both other connections originating in the dmPFC were moderately negative (AC and FFG). This condition also resulted in a decrease of connectivity from AC to the AG and a strong increase in connectivity from FFG to AG.

#### Incomprehensive content—E/iC

In the foreign language condition, connectivity from dmPFC was mildly negative to all other regions (AC, FFG, AG). Further, both the AC (weak) and the FFG (strong) exerted a positive connection on the AG.

## Discussion

We aimed at clarifying several aspects related to the temporal development of neural activation in regions that are responsible for processing emotionality in three communication channels in an experiment studying empathy with naturalistic stimulus material. Based on a reanalysis of a data set, these findings led way to an effective connectivity analysis in which we further sought to disentangle the relationships between these regions and an area responsible for processing multi-channel information and serving self-referential functions in the dorsomedial prefrontal cortex.

The FIR analysis of the three experimental conditions in which two channels were emotional and one was neutral resulted in significantly lower activations of the respective channel-related area compared to the fully emotional condition. The time-course of the neutral condition was in between the other conditions for the first 5–6 time-bins. After about 2/3 of the video clip duration (time-bin 7 for the AC, time-bin 8 for the FFG and for the AG) the activation in the respective bimodal condition surpassed that of condition N. Although this was statistically significant for the AC at time-points 8 and 9, and for the AG at time-point 9, it was observable that the bimodal condition changed from “below neutral” to “above neutral.”

This initially diminished activation (time-bin 1 to approx. 6 or 7) might have been the result of a suppression by other emotionally informed regions in order to inhibit the incongruent communication channel—or in other words—to reduce the prediction error reported by that region. As evident in Figure 1 while referring to the original analysis of this data-set (Regenbogen et al., 2012a), the whole-brain fMRI analysis showed that on average, bimodal conditions resulted—at least descriptively—in even less activation in the respective area (bimodal AC for E > E/nP (uncorr. *p* = 0.001), bimodal FFG for E > E/nF (uncorr. *p* = 0.005), and left AG for E/nC (n.s., *p* = 0.667)) than the fully neutral condition. In this reanalysis, we were able to disentangle the underlying time-course of the actual process to some degree. A prolonged presentation of the respective incongruent information may have then emphasized bottom-up processes which resulted in a stronger activation than the response observed in neutral videos toward the end of the stimuli. On a conceptual level, this could mean that although presented with incongruent information and thereby ambiguous (emotional and neutral) cues, contextual embedding still takes place to some degree, giving way to a successful late appraisal (De Vignemont and Singer, 2006) and empathy.

The general pattern of the *post-hoc* BMS, however, did not corroborate our hypotheses regarding this just-stated emphasis of bottom-up processes. One striking aspect of the graphical illustration of the DCM results (Figure 3) is the control that the dmPFC exerts on all other regions irrespective of the experimental condition. This pattern rather suggests a top-down control of the higher region dmPFC on the two lower regions (AC and FFG) as well as on AG. The fact that most of the coupling parameters (13 out of 18) have a negative sign may indicate a reduction of prediction errors reported by these lower regions. Remarkably, the condition transporting consistently emotional information (E) shows a unidirectional suppression of all three regions by the dmPFC, which might underscore the suppression of prediction error in the non-ambiguous emotional condition. The other likewise non-ambiguous but neutral condition (N), however, shows a synaptic gain in the connectivity from dmPFC to the two lower regions. As a rather speculative interpretation of this finding we may consider the neutral condition as being less informative so that this synaptic gain possibly reflects some cognitive effort in order to maintain attention in light of the rather boring content of the received message. Taking into account its documented role in self-referential processing (Wolf et al., 2010) and differentiation into self and other (Lamm et al., 2010), the dmPFC may—on a speculative note—not necessarily play a central role in fully neutral clips but rather in clips requiring some mental reappraisal of agency attribution.

Regarding the Bayesian brain hypothesis outlined in the introduction our results do not substantiate the idea that incongruent neutral information displayed by one out of three communication channels leads to a prediction error in the corresponding region at the lowest level (e.g., in FFG for E/nF or AC for E/nP) which then would be passed on to higher levels (dmPFC or AG). One reason for this lack of support is due to the rather bold assumption regarding the hierarchical ranking of the selected cortical regions: the AC and FFG at the lowest level, the dmPFC at the highest, and the AG somewhere in between. Another related argument pertains to the hierarchical distance between these regions which is assumed to be quite large, including polysynaptic pathways probably via several regions. This possibility raises the question if there are too many pathways and (cognitive) processes involved challenging the idea of a uniquely recordable hierarchy among the selected regions.

A recent meta-analytical review of vigilant attention proposed that the medial prefrontal cortex [including the dorsal anterior cingulate cortex (dACC)] and the anterior insula also belong to a network for “energizing” and performance monitoring (Langner and Eickhoff, 2012). While the dACC and the anterior insula are assumed to reactivate vigilant attention in cases of mind wandering when attention cannot be sustained, the medial prefrontal cortex (roughly corresponding to the dmPFC in the present study) withholds preplanned responses and maintains the intention and preparation to respond. The present results of the task-dependent connectivity of the dmPFC may point toward its role as an “energizer” of sensory modalities, allocating attention to functionally specialized, sensory regions. At least the ambiguous conditions lacking emotionality in facial expressions (E/nF) and speech content (E/nC) lead to an enhancement in the connectivity from the dmPFC to the corresponding region (FFG and AG, respectively). The consistently neutral condition resulted in a similar increase in the strength of connectivity from dmPFC to FFG and AC. This phenomenon may be explained by “inverse effectiveness,” where obscured, ambiguous or otherwise degraded information in a specific modality leads to an enhanced and thereby compensating allocation of resources on that modality (Senkowski et al., 2011; Stevenson et al., 2012). In the present study, the less clear or unique a source of information is, the greater is the need to take into account more available pieces of information and rely on those. Here, additional information is increasingly attended and used if information is obscured or ambiguous. Given the dmPFC's role in processing at least two channels (Regenbogen et al., 2012a), i.e., multimodal information, it seems plausible that it was involved in redistributing information flow in cases of ambiguity as well as increased effort to carry out agency distribution while it may have been harder to mentalize or empathize with someone who was displaying an ambiguous message. Although this interpretation might bear some plausibility, it reveals some limitations of the present study which will be discussed below.

Assuming that a crucial part of semantic processing is accomplished in AG, which roughly corresponds to Wernicke's area in our study, it is quite intriguing that the FFG → AG connection is always strongly enhanced by stimuli containing emotional facial expressions, while this connection is only moderately enhanced in the consistently neutral condition and even suppressed in condition E/nF. Although this might underscore the importance of visual stimuli and corroborate the Colavita visual dominance effect (Colavita, 1974), our results should be regarded as preliminary because of our assumptions about semantic processing and all restrictions that pertain to implicit or explicit assumptions in every DCM study, i.e., inclusion of brain regions or restrictions of connectivity structure.

### Limitations

As some of the interpretations already hinted to weaknesses and limitations of the present study these should be elaborated on in this section. Regarding the FIR time-course analyses it must be emphasized again that these should be regarded as exploratory. The aim of this analysis was to look for some differences in the time-course of selected modality-responsive regions of interest in respective conditions. Although the time-by-condition interaction was significant for these regions, one has to keep in mind that due to the relatively large degrees of freedom particularly for the factor TIME quite subtle differences in the time-courses may yield the interaction significant. Moreover, the *post-hoc t-tests* have been corrected only for the number of independent comparisons within each of the three regions, yielding these tests more liberal and therefore exploratory.

With respect to the analyses of effective connectivity it must be emphasized that model selection can be regarded as both boon and bane of neuroscientific progress. On the one hand, the model-based approach, where explicitly devised models compete against each other for describing and explaining the same data set, brings forth our understanding of the neuronal implementations of cognitive processes. Maybe model selection is better suited in comparing competing explanations than conventional null-hypothesis significance tests where one single model is tested against a usually uninteresting chance explanation. On the other hand, model selection procedures heavily rely on the validity of (the definition of) the model space. In other words, one has to assure that at least one reasonable good model is included in the model space where it is hard to decide if a specific space is broad enough and suitable models are included.

In a similar vein, the selection of brain regions in a neuroscientific approach using BMS (for fMRI) is crucial as model selection requires the same data to be explained by all models and a different choice of regions changes the data set. Additionally, more regions make the potential model space grow exponentially rendering an exhaustive model space impossible to deal with. In a recent study, the models to be compared included only four regions and three different inputs and though the searched space was based on constraints it nevertheless included more than 4000 models which was managed by a high-performance computing cluster (Kellermann et al., 2012).

Because of this computational burden we decided to apply a *post-hoc* BMS which searches a subspace of one “full” model without the need to invert each single model within the search space (Friston and Penny, 2011). Although such a greedy approach makes the search of a huge space feasible within several seconds it has been criticized because of combinatory explosion and therefore inclusion of many invalid models bearing the hazard of selecting such an invalid model purely by chance (Lohmann et al., 2012). Because of this risk, a greedy search without constraints should be characterized as exploratory and results as well as conclusions must be retained with care.

Contrary to the objections above one might also argue that the constraints we put on the model space (omitting connections between AC and FFG) excludes too many (plausible?) models *a priori*. Another argument refers to the number of regions being too little in order to adequately describe neuronal processing of multimodal emotional stimuli. For example, the “hierarchical gap” induced by restraining the data to a few regions may have resulted in a critical disregard of potentially available and valuable data. On the one hand, including intermediate levels may bridge that gap, but on the other hand it may severely impede putting reasonable constraints on plausible models and/or extremely complicates the problem of model space complexity.

Challenges for future studies therefore ask for both, inclusion of more regions (and thus more available data) which makes potential models more complex and at the same time putting reasonable constraints on the competing models permitting hypothesis-driven model selection. The antagonism between hypotheses-driven and exploratory studies remains, of course, but it might be regarded as a continuum wherein an individual study may be placed. As mentioned above, the present study should be regarded as an exploratory analysis of the effective connectivity among selected regions during multimodal processing of emotional stimuli.

## Conclusion

The best model, according to a greedy *post-hoc* Bayesian model selection, revealed a dynamic top-down connectivity from the dorsomedial prefrontal cortex (dmPFC) to at least two supposedly lower regions specialized for processing facial (fusiform gyrus, FFG) and auditory (auditory cortex, AC) stimuli as well as to a third region putatively involved in speech comprehension (angular gyrus, AG). The multimodal stimuli modulated these connectivities often in a sense that lacking emotional information in one of the three experimentally manipulated channels caused an increased input from the dmPFC to the respective channel-sensitive region. This pattern strengthens the suggested role of the dmPFC in executive functions like the allocation of cognitive resources and attention, as well as its role in mentalizing, empathy, and self-referential processing. While the experimental stimuli modulated all connections stemming *from* the dmPFC they also changed all connections *toward* the AG. Its property as a main receiving region might underscore its importance in the integration of information from different modalities in order to support comprehension. Notably in this regard are the couplings directed from the FFG to the AG as these were quite large in conditions in which the facial expression conveyed emotional information. These results indicate that the AG is possibly strongly supplied with emotional visual information which might be a hint toward another example of visual dominance.

Summarized, the results underlying the processing of complex dynamic stimulus with social relevance could be characterized in the temporal domain and show compensation effects driven by bottom-up effects in channel-sensitive regions potentially giving way to late appraisal of empathy-eliciting information. Further, the analysis of the dynamics between those regions and an important hub in social cognition and executive functions suggests a differential role in allocation attention toward incoming information depending on the emotional content and relevance of the stimulus, as well as the integration of multiple emotional and neutral modalities.

## Conflict of interest statement

The authors declare that the research was conducted in the absence of any commercial or financial relationships that could be construed as a potential conflict of interest.

## Footnotes

^1^Because contrasts did not only differ with respect to their emotionality, but also regarding ambiguity, all contrasts were analyzed in conjunction with E > N.

## Supplementary material

Table S1 ***Post-hoc* comparisons for each repeated measures ANOVA for the regions of interest**.
