# Supplementary material for: Connecting multimodality in human communication
Source: Front Hum Neurosci. 2013 Nov 8;7:754. doi: 10.3389/fnhum.2013.00754 (PMC3820976; doi:10.3389/fnhum.2013.00754)
Supplement: Table S1 — Post-hoc comparisons for each repeated measures ANOVA for the regions of interest. [file DataSheet1.DOC]

Supplementary Material Connecting multimodality in human communication

Christina Regenbogen^1,2^, Ute Habel^1,2^, Thilo Kellermann^1,2^*

^1^Department of Psychiatry, Psychotherapy, and Psychosomatics, Medical School, RWTH Aachen University, Germany. ^2^JARA Translational Brain Medicine, Jülich/Aachen, Germany.

Table S1. *Post-hoc* comparisons for each repeated measures ANOVA testing differences between three conditions each within each ROI. Bonferroni-correction was calculated for 16 (9-1)*(3-1) independent comparisons per ROI and yielded an adjusted alpha p-level of (1-0.025)^(1/16) = 0.001581.

| **Time bin** | **Contrast** | **T (df=26)** | **p** |
| --- | --- | --- | --- |
| **Auditory cortex** | |  |  |
| 1 | E>N | 3.465 | 0.002 |
| 2 |  | 3.341 | 0.003 |
| 3 |  | 1.541 | 0.135 |
| 4 |  | 2.587 | 0.016 |
| 5 |  | 5.117 | <.001* |
| 6 |  | 3.460 | 0.002 |
| 7 |  | 8.417 | <.001* |
| 8 |  | 6.774 | <.001* |
| 9 |  | 3.045 | 0.005 |
| 1 | E/nP>N | 3.149 | 0.004 |
| 2 |  | -1.758 | 0.090 |
| 3 |  | -1.894 | 0.069 |
| 4 |  | -1.702 | 0.101 |
| 5 |  | -1.491 | 0.148 |
| 6 |  | -1.505 | 0.144 |
| 7 |  | 2.890 | 0.008 |
| 8 |  | 7.889 | <.001* |
| 9 |  | 4.793 | <.001* |
| 1 | E>E/nP | 1.836 | 0.078 |
| 2 |  | 5.051 | <.001* |
| 3 |  | 3.640 | <.001* |
| 4 |  | 5.239 | <.001* |
| 5 |  | 7.106 | <.001* |
| 6 |  | 5.227 | <.001* |
| 7 |  | 2.859 | 0.008 |
| 8 |  | -0.044 | 0.966 |
| 9 |  | -1.553 | 0.133 |
| **Fusiform gyrus** | |  |  |
| 1 | E>N | .810 | 0.425 |
| 2 |  | -0.775 | 0.445 |
| 3 |  | 1.398 | 0.174 |
| 4 |  | 2.746 | 0.011 |
| 5 |  | 4.114 | <.001* |
| 6 |  | 4.304 | <.001* |
| 7 |  | 2.165 | 0.040 |
| 8 |  | 6.641 | <.001* |
| 9 |  | 3.635 | <.001* |
| 1 | E/nF>N | -0.015 | 0.988 |
| 2 |  | -1.055 | 0.301 |
| 3 |  | -0.885 | 0.384 |
| 4 |  | -0.824 | 0.417 |
| 5 |  | -0.991 | 0.331 |
| 6 |  | -0.619 | 0.541 |
| 7 |  | -1.227 | 0.231 |
| 8 |  | 1.348 | 0.189 |
| 9 |  | 3.436 | 0.002 |
| 1 | E>E/nF | .851 | 0.402 |
| 2 |  | .673 | 0.507 |
| 3 |  | 3.786 | 0.001 |
| 4 |  | 4.416 | <.001* |
| 5 |  | 5.775 | <.001* |
| 6 |  | 4.632 | <.001* |
| 7 |  | 3.643 | 0.001 |
| 8 |  | 4.834 | <.001* |
| 9 |  | .400 | 0.693 |
| **Angular gyrus** | |  |  |
| 1 | E>N | -0.348 | 0.731 |
| 2 |  | -0.544 | 0.591 |
| 3 |  | 1.246 | 0.224 |
| 4 |  | 5.084 | <.001* |
| 5 |  | 6.278 | <.001* |
| 6 |  | 6.311 | <.001* |
| 7 |  | 6.221 | <.001* |
| 8 |  | 5.955 | <.001* |
| 9 |  | 3.377 | 0.002 |
| 1 | E/nC>N | -0.622 | 0.540 |
| 2 |  | -0.377 | 0.709 |
| 3 |  | -1.060 | 0.299 |
| 4 |  | -0.585 | 0.564 |
| 5 |  | -0.035 | 0.972 |
| 6 |  | .254 | 0.802 |
| 7 |  | -0.027 | 0.979 |
| 8 |  | 1.969 | 0.060 |
| 9 |  | 4.256 | <.001* |
| 1 | E>E/nC | .276 | 0.785 |
| 2 |  | -0.164 | 0.871 |
| 3 |  | 1.999 | 0.056 |
| 4 |  | 4.608 | <.001* |
| 5 |  | 6.685 | <.001* |
| 6 |  | 6.738 | <.001* |
| 7 |  | 5.357 | <.001* |
| 8 |  | 4.284 | <.001* |
| 9 |  | -0.834 | 0.412 |
